# Can patients make heads or tails of enhanced primary health care (EnPHC)? Experience through their own journey

**DOI:** 10.1186/s12875-020-01254-2

**Published:** 2020-09-04

**Authors:** Mohammad Zabri Johari, Zalilah Abdullah, Ainul Nadziha Mohd Hanafiah, Nur Izzati Mohammed Nadzri, Siti Aisyah Razli, Yuke Lin Kong

**Affiliations:** 1 Institute for Health Behavioural Research, National Institutes of Health, Ministry of Health Malaysia, No: 1, Block B3, Jalan Setia Murni U13/52, 40170 Shah Alam, Selangor Malaysia; 2 Institute for Health Systems Research, National Institutes of Health, Ministry of Health Malaysia, No: 1, Block B2, Jalan Setia Murni U13/52, 40170 Shah Alam, Selangor Malaysia

**Keywords:** Patient, Enhanced primary health care, Non-communicable disease, Intervention

## Abstract

**Background:**

Implementation of the new Enhanced Primary Health Care (EnPHC) intervention aims to improve service quality and experience at primary healthcare clinics; especially to newly diagnosed patients. This was achieved by restructuring and improving existing services to better manage non-communicable diseases amongst patients. Objectives of this study are to explore patients’ experiences of the EnPHC intervention, to document their feedback and to determine effects of EnPHC intervention on patients.

**Methods:**

This phenomenological qualitative study focussed on patients’ experiences in relation to EnPHC interventions. Participants were purposely selected from a group of patients who attended the eight intervention primary healthcare clinics in Johor and Selangor regularly for treatment. Data collection was conducted between April to July 2018. Semi-structured interviews were conducted at average an hour per interview for four to five patients per clinic. Interviews were audio recorded, transcribed verbatim, coded and analysed using a thematic analysis approach.

**Results:**

A total of 35 patients participated. Analysis revealed five main themes about patient experiences receiving the EnPHC intervention. These are: (1) health assessment in disease progress monitoring, (2) patient-doctor relationship and continuity of care, (3) professionalism in service delivery, (4) ensuring compliance in achieving health targets and (5) communication skills. Each theme represents an important aspect of the service, how it should be delivered within the patient expectations and how it can improve patient’s health through their lens.

**Conclusion:**

Even though patients were not able to exactly identify the EnPHC intervention components implemented, they are able to describe the process changes that occurred; enabling them to improve their healthcare status. Engagement is necessary to better inform patients of the EnPHC intervention, its purpose, mechanisms, changes and importance for healthcare. It would reduce resistance and increase awareness amongst patients at the clinic.

## Background

Individuals newly diagnosed with a chronic disease often experience difficult situations due to lack of understanding of the disease they are having and the impact of daily activities on their health; leading towards a multitude of emotional reactions [1]. Individuals affected have to reassess daily activities like grocery shopping, cooking and eating meals or participating in social gatherings, and adjust their practices to the new demands of chronic disease management. Upon encountering new information and experiences, they quickly assess them to fit their existing understanding of the world [2]. This means, changes are necessary in order to fit new information, order of activity and priorities. Over the years, individuals are increasingly expected to be in charge of their own health and to be involved in self-health decisions due to the growing evidence that individual participation in disease management has several benefits such as increasing knowledge, increased satisfaction with treatment decisions and better treatment adherence [3–6].

In the process of patient decision making for treatment management, several factors such as personal values [7] their relationship with health professionals, their diagnosis and health status, the type of decision they need to make are regarded as associative factors. Several studies assess patients’ preferences towards involvement in medical decision-making which relates to preference of some patient groups to leave the decision to their physician, while most patients want to share decisions with their health professionals [6, 8].

Malaysia utilizes a mixed healthcare delivery system, which includes a universal government (public) healthcare and a private healthcare system, to ensure reasonable levels of physical access to healthcare services for the majority of the population [9]. The government healthcare is heavily subsidized and is subdivided into a three tier system - primary, secondary and tertiary care. Primary care focussed on community based preventive care with basic to intermediate care on the population and is the most diverse - with a coverage of a healthcare facility of 5 km radius that caters specifically for the rural population to allow ease of healthcare access. Secondary and tertiary care focussed on curative care with the latter more on intensive curative care [10]. The private healthcare system on the hand is divided into a dual system (primary and secondary) that is driven by a full-paying system through insurance or out of one’s own pocket [11].

In Malaysia, the increase of non-communicable diseases (NCDs) puts a heavy burden on primary healthcare. This raised a red flag suggesting the need for primary health care (PHC) service delivery improvements; responding to new challenges in the ever-changing health landscape. Since the 1980s, Malaysia has continuously attempted to improve the existing healthcare system. The Ministry of Health Malaysia has embarked on an improved primary care intervention since July 2017 called Enhanced Primary Health Care (EnPHC) initiative to improve NCD management for Malaysians at 20 primary care clinics in Johor and Selangor.

The EnPHC initiatives consisted of redesigning work processes in the clinic, community intervention through community enrolment and profiling and improvement of the referral system between clinic and hospital. One of the aims of EnPHC is to ensure improvements in patient care experience towards a patient-centred approach using active, population-level strategies for health and wellness. The intervention involve several transformations at the clinic which focused on five areas of achievement; to which three are assessed at patient experience - emotional, quality and efficiency needs [12].

This study was done to assess the effectiveness of implementation of the EnPHC intervention from the patient’s perspectives. It is part of a larger study to evaluate the implementation process. This paper focuses on the patients’ perception and experience with the healthcare services during the implementation period.

### Objective

The objectives of this study are to explore patients’ experiences in making sense of the improvised services, to document patients’ feedback for improvement of the interventions and to excerpt patients’ perception and experience in receiving services during the intervention period.

## Methods

### Design

This is a phenomenological qualitative study conducted to explore patients’ experiences with the EnPHC intervention; focusing on the intervention characteristics that they can identify, give feedback and suggestions to improve the intervention for future implementation.

### Setting

EnPHC intervention was conducted at 20 primary healthcare clinics in rural and urban areas in Johor and Selangor. Participants for this qualitative study were recruited from eight of these clinics. These clinics were purposely matched for both States in terms of clinic size, capacity and locality.

### Intervention

**Fig. 1:**
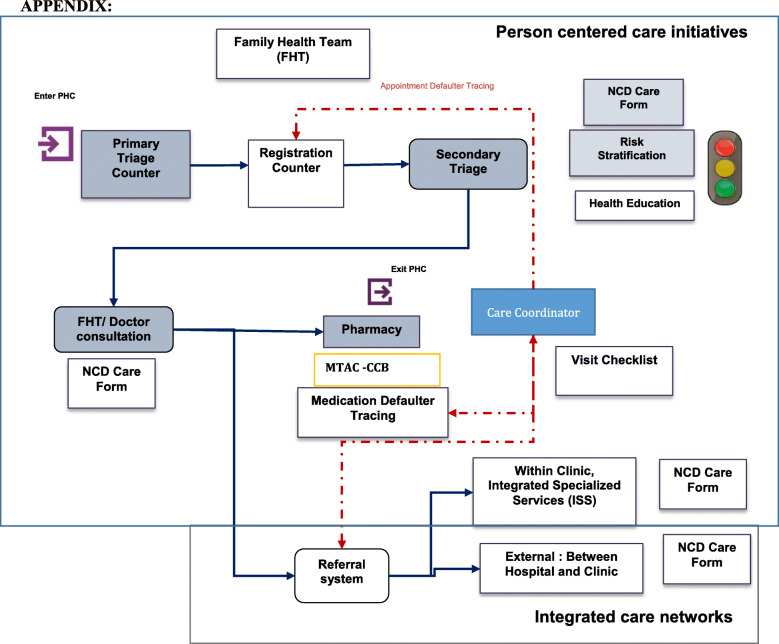
Flow process at the clinic and complex, integrated intervention package of the EnPHC implemented at every stage of process flow patients will undergo

The EnPHC intervention is dubbed as an introduction of a complex intervention packaged - it introduces and improves many existing mechanisms in the existing healthcare delivery system. The main intervention components in the EnPHC intervention included the establishment of community interventions through fostering population enrolment and population risk profiling for early NCD risk management and case detection, deployment of a two-tiered triaging system with multiple sub-components in the primary healthcare clinics, introduction of the Care Coordinator role in the primary healthcare service, improvements to the NCD care management, particularly on the aspect of service delivery and documentation, and improvements to the internal and external referral system [12, 13]. The intervention package changes the existing set up at the clinic to a more structured, detailed and improved process-flow of healthcare delivery which comprises two parts – the person centred care initiatives and the integrated care network as described in Fig. 1.

### Participants

Purposive sampling was used to recruit participants for this study. Participants were registered patients who are Malaysian, attended regular follow-up appointments for at least 2 years at the site clinic, or attended at least three clinic (NCD) appointments during the EnPHC implementation period. Excluded patients are individuals less than 18 years and those with reduced mental capacity. Patients were approached for interviews by the clinic Care Coordinators who identified patients based on the criteria above. Patients had no contact with the researcher prior to the study and were briefed about the study concept and process before consent was taken. Data saturation guided participants’ recruitment and saturation was achieved at patient 32 but we have added an additional 3 patients to ensure no new information detected. All participants approached agreed to be interviewed.

### Data collection

Interview data analysed in this study was collected as part of the EnPHC: Process Evaluation (EnPHC: PE) study where patients were interviewed at the end of the ten months intervention, between April until July 2018. Interview questions were developed for this study were based on Karl Weick’s Sense-Making Theory (SMT) Framework [14–16] and were pre-tested on a group of random patients in a public clinic [ADDITIONAL FILE – Interview Questions]. This framework was chosen to create the interview guide for the participants - the questions’ core explored changes perceived by the patients during intervention implementation. Questions format were developed with specific prompting questions to elicit experience from patients as they entered the clinic and try to make sense of every intervention that they could identify, and explore the details of their experiences. Interview sessions were conducted face-to-face by the research team members who were trained in qualitative methods and were not close acquaintances with any of the participants to avoid potential response bias.

Participant information sheet and informed consent was given to participants prior to interviews. Each in-depth interview (IDI) was conducted at the clinic in a quiet, secure and comfortable room; lasting between 30 to 120 min and were audio-recorded, noted in fieldnotes and transcribed verbatim. Confidentiality was ensured where participants’ identifiers were removed from transcripts. Each transcript was cross-checked by the research team through audio listening and usage of field notes.

### Rigor & Trustworthiness of the study

The measure of rigor and trustworthiness in this study follows four criteria established by Lincoln and Guba [17].

#### Credibility

Comprehensive field notes were kept during the data collection process that included a record of gestures and other nonverbal cues by participants. Participants were also asked to confirm data obtained in through interview process. This is done through confirmation of feedback given periodically as the interview progresses. We also ensure the principle of theoretical saturation.

#### Confirmability

Confirmability of data was ensured through verbatim transcription of interview records and data triangulation.

#### Dependability

The adequacy of the study protocol was assessed by two senior researchers who were not part of the study. A comprehensive description of the study setting, details of the study design and, technique for data analysis are all provided.

#### Transferability

We provide detailed information for potential transferability, including description of the study, the methodology and the participants.

### Analysis

Data were analysed using a thematic analysis method done by the EnPHC: PE research team members who are experts in their own field of expertise [13]. First, researchers read transcribed interviews to identify preliminary themes independently. The analysis took a realistic interpretivism approach where participant experiences were interpreted and coded in line with existing information in the EnPHC guidelines to match their experiences without making assumptions from the feedback given [18, 19]. Next, meaning units were reviewed, identified, and sorted into themes before classified into subgroups. Lastly, through consensus and data saturation, contents of each code group was summarized and categorized into main themes based on participant feedback. Best representative quotes for each theme were chosen to support results. New emerging themes based on participant feedback were coded, arranged and presented, and were coded independently and presented accordingly.

## Results

A total of 35 patients were recruited via purposive sampling at eight clinics sampled from a total of 20 intervention clinics in Selangor and Johor. Participant characteristics were presented in Table 1. Participants’ age ranged from 25 to 76 years old, with the mean age of 57 years old; slightly more than half were females; almost all were Malay and majority had secondary level education. Participants varied in their work history. Some were unemployed or housewives, retirees and self-employed, and some are working in private or public sectors. Participants were diagnosed with diabetes from as early as 1986 to the most recent diagnosis in 2018, and had an average of three to four visits to the clinic since the implementation of the EnPHC intervention in July 2017.

**Table 1:** Details of IDI participants

Characteristics	Number of Participants
**Age groups**	
Below 40	1
40–49	8
50–59	8
60–69	11
70–79	6
**Gender**	
Male	15
Female	20
**Ethnicity**	
Malay	32
Chinese	1
Indian	1
Iban	1
**Education background**	
Academic degree	4
Secondary high school	17
Primary school	14
**Job**	
Housewife	4
Retiree	12
Employed	10
Unemployed	9
**Appointment frequency since July 2017**	
3	14
4	12
5	4
6	4
7	1

*Note: All participants are Diabetic and/or Hypertension patients*

**Fig. 2:**
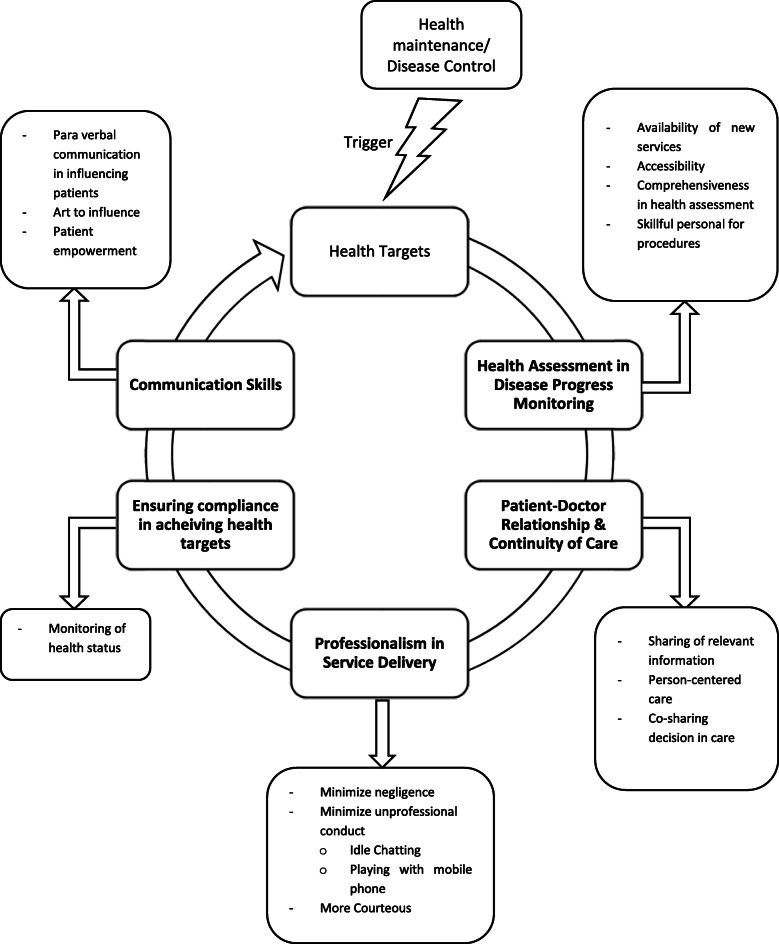
Emerging themes from derived perceived health status affected by EnPHC intervention presented in a health status maintenance/disease management cycle

Thematic analysis from the qualitative interviews revealed five major themes depicting the process of maintaining patients’ health status or management of their diseases via experiencing the EnPHC intervention. Through themes presented in Fig. 2, participants experiencing the EnPHC intervention package would have made health assessments in disease progress monitoring, to allow them to set their health targets using the EnPHC intervention. They also would understand the patient-doctor relationship process and the continuity of care they received. Furthermore, participants would praise the professionalism of healthcare providers in providing service delivery. At the same time, they understand the process of ensuring compliance to achieve the health target set earlier with their healthcare providers (HCP) and also understand the necessity of communication skills in achieving their target.

### Health assessment in disease Progress monitoring

Participants were able to identify new services or additional health assessment available under the EnPHC intervention despite most participants claimed to have not seen any information or have been informed of the EnPHC interventions. Participants noted services previously inaccessible or having the need to travel to access them are now available at the intervention clinic. Through the EnPHC intervention implementation, the services are now within reach at their own registered clinic. This allowed better accessibility to participants and in turn improved adherence to their disease management. Participants have also noticed that health assessments are more comprehensive than before.

“*Eye-examination is now every 6 months; not once a year and it’s done here (at the clinic) not like before at* ***K*** *(at a different town). It is closer now; easier now and we have to check for everything. Heart, heart checks, etc*” (P7, Female, 46 years old).

Participants also noted that HCP conducting health assessments should be skilled in technical knowledge and procedures. While some complimented HCPs capabilities in providing good knowledge transfer to patients for disease management, others were not particularly happy with the lack of technical knowledge that HCPs should have shown in their service.

“*He should have told me ‘uncle, you can eat this or that but more often (in small amounts). Instead he said ‘you have to eat more often’. I would have gone hyper and overdosed (with sugar) if I had listened! In terms of knowledge, that doctor is lacking. He may only have the theoretical knowledge but not practical ones!*” (P3, Male, 58 years old).

### Patient-doctor relationship and continuity of care

A few themes were highlighted that were very pertinent for the continuity of care which foreshadows success in EnPHC patient care. First and foremost, to the participants, sharing of information was vital especially if the information directly concerns participants health or disease status. They greatly appreciated HCP provided advice and felt the relationship they had was greatly beneficial in the management of their disease.

“*Previously I had seen different doctors … now I only see Dr M … I am very happy because I saw good results for my treatment, he even congratulated me … he also said we will see each other in the next four months … I feel so excited!*” (P27, Male, 59 years old).

Participants felt “cared-for” when HCP would focus their attention towards their care thus the emergence of the third theme – patient-centred care. They believed they felt appreciated that they are cared for in a special manner by the HCP. This special treatment was further enhanced with the focus of care and sharing of information, co-sharing for decision making is also important to the continuity of care. This sharing of information is felt as a special treatment that was not present prior to the implementation of EnPHC.

“*He (the doctor) advises me a lot – he told me there’s no difference between tobacco rolled cigarettes and normal cigarettes – they’re both bad! He advised me to cut down on smoking, red meat and so forth. He told me to eat more fish.* (P6, Male, 51 years old).

### Professionalism in service delivery

In delivering health care service; professionalism is paramount. Three important sub-themes were identified clearly – negligence reduction, professional conduct and courtesy. Patients felt that attempts in minimizing negligence should be prioritized as they felt the service would be severely disrupted if HCPs do not act professionally. As clients; patients have a certain expectation of how HCP should act and behave and to the patients; poor behaving HCP reflects the service as a whole.

“*When you talk harshly to patients, that’s why the community does not want to come for your services. And then you ask why people don’t come for your services, it’s because you’re causing difficulty, you talk harshly to us, or you are not happy with something*” (P16, Male, 70 years old).

Participants also mentioned that unprofessional conduct should be minimized to reduce resentment towards HCPs; more so towards counter staff at the clinics. As there is a perceived high volume of patients coming to the clinics, patients believed in the optimization of counter service and its manpower. Patients perceived lack of service provision quality at the counter were due to idle chatting or playing with mobile phone – this increases delay in the queue; and can be a reason for anger.

“*Look at those counters – they’re enough! But the staff there are chitter chatting! I see they’re not doing work and I have (been) waiting like forever. I almost wanted to tell them off; I am angry and I’m not happy*” (P35, Male, 58 years old).

“*When you have clients, work; don’t hold your handphone*” (P26, Female, 46 years old).

Above all, participants were expecting HCPs that can be courteous to them as it is the essence of professionalism in healthcare delivery and even though courtesy is not present in all HCPs, those that did present the traits were duly remembered by the participants.

“*Alhamdulillah, I’ve met one lady doctor and she explained to me everything – my diabetes, my high blood pressure, my kidney problem, my cholesterol. I am perfectly happy with everything*” (P29, Male, 70 years old).

### Ensuring compliance in achieving health target

In order to meet their health targets, patients require assistance to comply in achieving their health targets. With regards to this, participants perceived the process of health status monitoring is crucial. Participants also mentioned having experienced monitoring in the EnPHC intervention through counselling at the clinic pharmacy on medication and healthy eating.

“*Last time when my mother took medication they didn’t tell (her) what to do but now they explained to me how to take proper medication and diet*” (P5, Female, 50 years old).

However, there was negative feedback from participants, where lack of skilled manpower supposed needed to provide the necessary services is seen as a lack to the success of the EnPHC intervention.

“*The doctor is giving theoretical information that doesn’t make sense. They only theory, not the practical knowledge that they should tell us*” (P35, Male, 58 years old).

### Communication skill

Finally, in completing the cycle, participants noted emphasis on communication skill. Paraverbal communication includes tone, facial language and methods of communicating ideas, information and concepts. A staff communicating in a soft and polite manner was perceived to have resulted in a pleasant experience and vice versa.

“*The nurse communicated to us in a soft manner and we were pleased with the experience. The communication towards the clients here (clinic) were not as rude as compared to the General Hospital*” (P11, Female, 45 years old).

“*If you (doctor) communicate in a harsh manner, people would be displeased. My friend even got mad at the doctor*” (P35, Male, 58 years old).

## Discussion

Participants in the EnPHC intervention clinics were not able to infer the exact interventions implemented mostly due to the lack of awareness of the EnPHC interventions; however, they were able to perceive the workflow and part of the process – even as much as identifying gaps in the intervention implemented. Participants were also able to act on their own health based on the interventions process implemented and make sense of the effects it had on their health.

The five themes derived from the their journey through the EnPHC intervention have revealed health education is more comprehensive than before but require assistance in achieving their health targets. This aligns with previous study findings where health education appeared to be one of the factors affecting NCD preventive actions [20, 21]. Health education is perceived as outstandingly important to patients in studies by Garcia-Perez and Ahmad; as HCP can offer more by explaining self-health-monitoring or self-management of NCD that are highlighted as beneficial by patients. Moreover, information received, education, and motivation by HCPs leads to improved adherence towards medication and treatment which consequently will provide better health outcomes [22, 23]. According to another study, patients lack confidence to manage their health by themselves and they are unsure how to modify their lifestyle to curb NCD. Therefore, they require detailed information and more time to engage with HCPs [24]. A study in Africa found out that individual counselling is the major factor that contributes to compliance with treatment [25].

Patients from this study also noticed continuity of care as HCPs demonstrated consecutive follow up through interventions introduced. According to previous findings, patients would like to acquire more enlightenment on their current health conditions and avoid any complications [26, 27]. Such follow-ups impacted adherence to treatment in a good way according to a study in France [28].

From the findings, patients also perceived services are more accessible after implementation of EnPHC which in accordance to study demonstrated by Goldthorpe [29] where patients are in favour of appointments and treatments at clinics near to them. The paper also discussed a lot of interpersonal skills of HCPs as in line with the study findings - proficient communication is vital for patients to have the urge to make an effort for self-monitoring and be engaged in overcoming their own NCD issues. Although most HCPs are equipped with hard skills (technical skills and theoretical knowledge) some lack soft skills (interpersonal attributes or people skills) [30]. Soft skills mentioned include empathy, building trust and relationship, giving complete and precise information, providing non-judgemental environment for patients to be able to ask questions without hesitation or hiding any important information during treatment, ability to calm down anxious patients, and others. Patients are in favour of good interpersonal relationships with HCPs which facilitates long term care. Negative mannerism in HCPs discouraged patients to be open and inhibit trust building. A trust-based relationship leads to better clinical outcomes and patients are more likely to adhere with treatment plans. A study found patients with common cold had a shorter duration of illness when they perceived HCP as being empathic which triggers the immune system changes [31, 32].

Therefore, interpersonal skills and communication skills needed to be assessed among HCPs as it affects the emotional well-being of patients and their quality of life positively which consequently leads to better health outcomes. According to a study in Spain, poor communication causes poor compliance towards treatment which will affect health outcomes [31]. HCPs should convey information to patients in the way that is contextualized according to the needs and situations of the patients and ensure manpower focus is more patient-centred and committed at every counter or room. Comprehensive training programs to instil patient-centred communication skills should be innovated to increase HCPs confidence level and self-assessed communication skill that will contribute to patient satisfaction. This is especially important during medical students development to bridge the gaps between their ability and their “unconscious competence”. It is also highly needed to ensure future medical doctors are provided with good, safe patient care across a broad range of competencies [33]. Besides that, patient survey and peer feedback should be incorporated as a method of overseeing patient-centred communication and the progress towards improving those skills [26, 27]. Policy makers should consider developing assessment on HCPs’ interpersonal skill by studying the essentiality of interpersonal skill and ways to develop it. Another finding suggested patients should be used as ambassadors that can influence and inform other patients in their communities through their own health conditions on how to self-manage NCD [34].

### Strength and limitation

Qualitative research methodologies are increasingly more accepted in the explorations of patients’ behaviours. In this study, the one-on-one interviews approach allows detailed exploration in individual perceptions of the EnPHC interventions – understanding the concepts and the implementation. These perceptions are not affected by the HCPs – allowing unbiased feedback from the participants to the researcher. This was achieved through cues and probing questions; further enhanced by the skills of the researcher. With most clinics selected being in rural areas and are mostly Malay centric; it was not surprising most participants were with secondary education level and due to the NCD reported; most affected are from the older population. This coincides with the National Health Morbidity and Mortality (NHMS) burden of disease data where most sufferers of NCD are largely from the suburban areas and of the older population [20, 21]. Although the numbers of participants are considerably small in comparison to quantitative studies; it was sufficient in providing saturated feedback on prevailing discussed issues at all 20 clinics. As such, it can be concluded that similar issues would crop up should the EnPHC implementation be upscaled to other localities. However, the level of honesty presented often thread towards emotional feedback and researchers had to thread carefully to avoid outbursts of emotions that may deviate from the original intentions of the questions asked. This may result in some questions or probing of questions that could not be completed. Furthermore, there were factors that were not accounted for in the study – family/close relations feedback and support in relation to the patient’s behaviour. It would be suggested exploration to these additional groups be conducted for future studies.

## Conclusion

Participants are able to make sense of the changes in process through their experience despite not being able to specifically identify the interventions of EnPHC. They are also capable of giving critical feedback based on their observations and interactions with the HCPs. Most importantly is they are able to make sense of the impact of the EnPHC interventions on their health improvements. In the possible future of further upscale of EnPHC interventions, it would be worth engaging the patients of the clinics on the details of the EnPHC interventions as it can increase the levels of awareness and improve understanding of the interventions itself.

## Supplementary information

**Additional file 1.** Interview Question. Description: Interview questions used for the study. The questions are listed as main questions and are dynamic in nature to allow further explorations based on participant feedback. The interview guide was developed using Karl Weick’s Sense-Making Theory as per described in the main manuscript.

**Additional file 2.** COREQ Checklist. Description: Checklist for publication of qualitative manuscript.

## Data Availability

The dataset that supports the findings of this article belongs to the EnPHC study. At present, data are not publicly available but can be obtained from the authors upon reasonable request and with the permission from the Director General of Health, Malaysia.

## Abbreviations

EnPHC: Enhanced Primary Health Care
NCD: Non-Communicable Diseases
PHC: Primary Health Care
EnPHC:PE: Enhanced Primary Health Care: Process Evaluation
IDI: In-Depth Interview
HCP: Health Care Provider
FHT: Family Health Team
MTAC-CCB: Medical Therapy Adherence Therapy-Cardiovascular Care Bundle
ISS: Integrated Specialised Services
