# Supplementary material for: Can patients make heads or tails of enhanced primary health care (EnPHC)? Experience through their own journey
Source: BMC Fam Pract. 2020 Sep 4;21:182. doi: 10.1186/s12875-020-01254-2 (PMC7487683; doi:10.1186/s12875-020-01254-2)
Supplement: Supplementary file 1 — Additional file 1. Interview Question. Description: Interview questions used for the study. The questions are listed as main questions and are dynamic in nature to allow further explorations based on participant feedback. The interview guide was developed using Karl Weick’s Sense-Making Theory as per described in the main manuscript. [file 12875_2020_1254_MOESM1_ESM.docx]

**Interview Questions:**

1. Are you aware of the Enhanced Primary Healthcare Intervention at your clinic?

Probe: Can you describe where do you see this information?

1. Are you aware of the changes happening in the clinic? Kindly describe these changes as you enter the clinic till you leave.

Probe:

a. Front entrance (Primary Triage)

b. Registration

c. Secondary Triage

d. Doctor’s Consultation Room

e. Pharmacy

f. External services (Nutrition, Patient Education, Others)

g. Other notable differences

All probes in Question 2 should include the following questions:

- Do they see the difference compared to 10 months ago or before intervention?
- Do they understand the purpose of this new “service”?
- Do they see the changes as beneficial or causing difficulty?
- Describe the changes structurally, changes in process/service, manpower, others.

1. Have you talked about the changes with anyone else (family, friends, colleagues)? What are their thoughts on the changes?
2. What are your expectations on the changes? How do you feel the services can be made better?
