# Supplementary material for: Can patients make heads or tails of enhanced primary health care (EnPHC)? Experience through their own journey
Source: BMC Fam Pract. 2020 Sep 4;21:182. doi: 10.1186/s12875-020-01254-2 (PMC7487683; doi:10.1186/s12875-020-01254-2)
Supplement: Supplementary file 2 — Additional file 2. COREQ Checklist. Description: Checklist for publication of qualitative manuscript. [file 12875_2020_1254_MOESM2_ESM.docx]

**Title : *Can patients make heads or tails of Enhanced Primary Health Care (EnPHC)? Experience through their own journey***

**Part 1: Based on COREQ (Consolidated criteria for Reporting Qualitative research) Checklist**

| **Topic** | **Item No.** | **Guide Questions/Description** | **Reported on Page No.** |
| --- | --- | --- | --- |
| **Domain 1: Research team and reflexivity** | | |  |
| ***Personal characteristics*** | | |  |
| Interviewer/facilitator | 1 | Which author/s conducted the interview or focus group? | MZJ, ANMH & ZA |
| Credentials | 2 | What were the researcher’s credentials? E.g. PhD, MD | MZJ: PHD (Newcastle), ANMH: MPH, KYL: BN, NIMN: Msc. (CIE), SAR: BSc (Bio), ZA: MB BCH BOA |
| Occupation | 3 | What was their occupation at the time of the study? | MZJ: Health Education Officer (Research), AMNH & ZA: Medical Officer (Research), KYL: Nurse (Research), SAR & NIMN: Research Officer |
| Gender | 4 | Was the researcher male or female? | MZJ: Male, AMNH, KYL, SAR, NIMN & ZA: Female |
| Experience and training | 5 | What experience or training did the researcher have? | Line 153-155 |
| ***Relationship with participants*** | | |  |
| Relationship established | 6 | Was a relationship established prior to study commencement? | No |
| Participant knowledge of the interviewer | 7 | What did the participants know about the researcher? e.g. personal goals, reasons for doing the research | No, Line 141-143 |
| Interviewer characteristics | 8 | What characteristics were reported about the interviewer/facilitator? e.g. Bias, assumptions, reasons and interests in the research topic | Line166-167 |
| **Domain 2: Study design** | | |  |
| ***Theoretical framework*** | | |  |
| Methodological orientation and Theory | 9 | What methodological orientation was stated to underpin the study? e.g. grounded theory, discourse analysis, ethnography, phenomenology, content analysis | Line 168-171 |
| ***Participant selection*** | | |  |
| Sampling | 10 | How were participants selected? e.g. purposive, convenience, consecutive, snowball | Purposive sampling, Line 135-139 |
| Method of approach | 11 | How were participants approached? e.g. face-to-face, telephone, mail, email | Approached by Care Coordinator, Line 139-141 |
| Sample size | 12 | How many participants were in the study? | 35 participants, Line 178-179 |
| Non-participation | 13 | How many people refused to participate or dropped out? Reasons? | None |
| ***Setting*** | | |  |
| Setting of data collection | 14 | Where was the data collected? e.g. home, clinic, workplace | At the clinic, Line 160-161 |
| Presence of non-participants | 15 | Was anyone else present besides the participants and researchers? | No |
| Description of sample | 16 | What are the important characteristics of the sample? e.g. demographic data, date | Line 178-186, Page 24 Table 1 |
| ***Data collection*** | | |  |
| Interview guide | 17 | Were questions, prompts, guides provided by the authors? Was it pilot tested? | Line 148-155, questions were piloted on random individuals to ensure comprehension and feedback. |
| Repeat interviews | 18 | Were repeat interviews carried out? If yes, how many? | No |
| Audio/visual recording | 19 | Did the research use audio or visual recording to collect the data? | Line 160-162 |
| Field notes | 20 | Were field notes made during and/or after the interview or focus group? | During and reviewed after interview, Line 160-162 |
| Duration | 21 | What was the duration of the inter views or focus group? | Ranging between 30-120 minutes, Line 160-162 |
| Data saturation | 22 | Was data saturation discussed? | Yes, Line 143, 172-174 |
| Transcripts returned | 23 | Were transcripts returned to participants for comment and/or correction | No, however each transcript was checked by separate researcher by listening to the audio record to ensure data accuracy. Line 167-176 |
| ***Domain 3: Data Analysis & Findings*** |  |  |  |
| ***Data analysis*** |  |  |  |
| Number of data coders | 24 | How many data coders coded the data? | Four |
| Description of the coding tree | 25 | Did authors provide a description of the coding tree? | Line 168-176 |
| Derivation of themes | 26 | Were themes identified in advance or derived from the data? | Themes were derived from the data |
| Software | 27 | What software, if applicable, was used to manage the data? | Microsoft Word and Excel |
| Participant checking | 28 | Did participants provide feedback on the findings? | No |
| ***Reporting*** |  |  |  |
| Quotations presented | 29 | Were participant quotations presented to illustrate the themes / findings? Was each quotation identified? e.g. participant number | Yes, they have been separated from the theme description to made clear and numbered based on participant quotation. Line 215-217, 223-226, 233-236, 243-245, 253-256, 262-264, 265, 269-271, 278-279, 283-284, 290-292, 293-294. |
| Data and findings consistent | 30 | Was there consistency between the data presented and the findings? | Yes, data presented in the form of themes and supported by quotations. |
| Clarity of major themes | 31 | Were major themes clearly presented in the findings? | Yes, each major themes were presented in each sub-headings. |
| Clarity of minor themes | 32 | Is there a description of diverse cases or discussion of minor themes? | Yes, Line 293-369 (Discussion) |

Acronyms: MZJ (Mohammad Zabri Johari), ZA (Zalilah Abdullah), ANMH (Ainul Nadziha Mohd. Hanafiah), KYL (Kong Yuke Lin), SAR: Siti Aisyah Razli
